# Treatment of Tetanus

**Published:** 1878-05

**Authors:** 


Treatment of Tetanus.—According to Mr. de Renzi (of Genes), the following is the proper procedure in the treatment of a case of tetanus:

1. Place the patient in a perfectly dark room, and only open the door partially once in four hours, to pass in food and drink.

2. Stop up the external ears with wax, and recommend the patient to rest as tranquilly as is possible for him.

3. Every hour give him out of a suitable vessel, soup, an egg, and two spoonfuls of white wine. For drink, water, with a very small quantity of wine.

4. To quiet pain give a little belladonna and ergot.

5. Have a carpet on the floor of the room. *Gaz. Med. de Paris* (*Gaz. des Hopitaux*, No. 123, Oct. 3, 1877.)—*Journal of Nervous and Mental Disease.*

———:o:———

The *Popular Science Monthly* publishes the following: A square metre of wall of a surgical ward in the Paris Hôpital la Pitié was washed, an operation that had not been performed during two years previously, and the liquid wrung out of the sponge was immediately examined. It contained micrococci in abundance, some micro-bacteria, epithelial cells, pus globules, and ovoid bodies of unknown nature. The sponge used was new, and had been washed in distilled water.

———:o:———

Several persons in Berlin, Germany, fell seriously ill after partaking of American dried beef which is sold there in tin cans. A chemical examination of the beef showed that the layer next to the lid of the can was strongly saturated with the salts of lead. The soft solder by which the cover was fastened was put on the inside so thickly that it came in contact with the meat and impregnated it with lead.

———:o:———

According to a statement in the Academy two healthy men allowed their limbs to be coated with impermeable plasters while the trunk was varnished with several layers of flexible collodium, which were allowed to remain for a week. None of the evil consequences observed in animals, made their appearance; there was no fall of temperature, no albuminuria, no exhaustion, no dyspnœa, convulsion or paralysis.—*Chicago Medical Journal.*

